# Joint and individual effectiveness of galvanic cutaneous stimulation and tactile stimulation at decreasing Simulator Adaptation Syndrome

**DOI:** 10.1371/journal.pone.0240627

**Published:** 2020-10-15

**Authors:** Germán Gálvez-García, Javier Albayay, Fernando Fonseca, Claudio Bascour-Sandoval

**Affiliations:** 1 Departamento de Psicología, Universidad de La Frontera, Temuco, Chile; 2 Département de Psychologie Cognitive & Neuropsychologie, Institut de Psychologie, Laboratoire d’Étude des Mécanismes Cognitifs, Université Lyon 2, Bron, France; 3 Dipartimento di Psicologia Generale, Università degli Studi di Padova, Padova, Italy; 4 Carrera de Kinesiología, Facultad de las Ciencias de la Salud, Universidad Autónoma de Chile, Temuco, Chile; 5 Departamento de Medicina Interna, Universidad de La Frontera, Temuco, Chile; University of Minnesota, UNITED STATES

## Abstract

This research was focused on investigating the effectiveness of galvanic cutaneous stimulation and tactile stimulation jointly and individually at mitigating Simulator Adaptation Syndrome. Forty drivers (mean age = 23.1 ± 3.4 years old, twenty women) participated in a driving simulation experiment. Total scores of the Simulator Sickness Questionnaire, head movements (an index of body balance), and driving performance variables were compared across four different stimulation conditions: i) baseline (where no stimulation was presented), ii) galvanic cutaneous stimulation and iii) tactile stimulation deployed individually, and iv) both techniques deployed jointly. The results showed that both techniques presented in conjunction alleviate Simulator Adaptation Syndrome and improve driving performance more effectively than when they are presented in isolation. Importantly, reduced head movements were only revealed when galvanic cutaneous stimulation was applied. We concluded that the reduction of this syndrome is due to an improvement of body balance (elicited by galvanic cutaneous stimulation), and a distraction from the symptoms (elicited by tactile stimulation). We encourage the use of both techniques simultaneously to decrease Simulator Adaptation Syndrome.

## Introduction

Motion sickness (MS) is a condition generated due to the perceptual difference between the expected and the actual motion. This condition can cause fatigue, cold sweat, disorientation, drowsiness, vomiting, among other symptoms [1]. Simulator Adaptation Syndrome (SAS), or simulator sickness, is a form of motion sickness experienced in virtual simulators. SAS is associated with more oculomotor and head symptoms such as headache, dizziness, and eyestrain [2, 3] Most people suffer from this syndrome to a greater or lesser degree [4], even reaching dropout rates of 30% in simulator studies [5].

The aetiology of these phenomena has been explained from several theories. Two main theories stand out from the rest. The sensory conflict theory [4, 6], postulates that SAS symptoms result from a mismatch between the visual, vestibular, and/or somatosensory systems. The discomfort is created by tuning to multiple streams of conflicting information among afferent sensory systems, interrupting the integration of expected information according to the typical template processed by the central neural system. For example, in a simulator the visual stimulus indicates self-motion, which conflicts with the vestibular system because it did not signal body motion. Importantly, this theory emphasizes that SAS is not produced by a simple mismatch among the senses. It further highlights the symptoms reflect the failure to integrate sensory input with predicted activity, formed by past patterns of sensations which accompanied motor activity failing to predict the current sensations accompanying similar motor activity. Thus, the crucial factor in the genesis of SAS is the sensory conflict and the discrepancy between the current and the expected sensory patterns. In this avenue, repetitive expose to SAS produces a sensory rearrangement which leads to a decrease in SAS symptoms. This theory has been extended and completed over time with a mathematical model [6, 7] and subjective vertical theory [8]. In this last one, for example, the spatial orientation is capital to estimate our own motion and orientation and with regard to the Earth’s vertical. Thus, the key sensory conflict in SAS is related to the conflict between the several ongoing sensations of the vertical and the organism’s internal model of subjective vertical based on past experience. From a different avenue, the theory of postural instability [6] offers an alternative explanation for the genesis of SAS. It states that SAS is caused by an inability to maintain a “stable” pattern of movement, whether this stability is slowly degraded or outright lost. Riccio and Stoffregen [9] postulated that sickness is due to an interference of the movements produced by imposed motion and the normal balance of the body. This interference increases the difficulty of maintaining posture stability, producing SAS symptoms. Several studies have supported that postural instability is correlated with MS [10, 11]. For instance, Merhi et al. [12] report that video gamers who performed more frequent head movements experienced greater MS symptoms. In another study by Bonnet, et al. [13], observers were exposed to low frequency movements in a moving room while standing. The results showed that there were more changes in body sway in the group that suffered from more MS symptoms compared to the group with weak symptoms. It is concluded that increased difficulty in postural stability is the cause of MS. Additional research supported that different body sway strategies are handled to stabilize postural control as a function of floor area requirements (e.g., land or sea; [14]). See [15] for a full description of SAS theories.

Different approaches have been tested to decrease MS and SAS symptoms. Beyond some drugs that, although effective at decreasing MS, can produce fatigue among other symptoms [16], behavioral approaches have been proved as suitable solutions to mitigate MS, such as biofeedback and cognitive methods [17, 18], and pleasant and relaxing music or smell for SAS [19, 20] among others. A countermeasure has proved to be especially effective in alleviating SAS, namely galvanic cutaneous stimulation (GCS). This technique delivers a short electrical impulse below the motor threshold and stimulates superficial skin nerve fibres of the neck muscles (usually the sternocleidomastoids) approximately 3–4 cm below the mastoid process. This area has a high density of subcutaneous sensitive fibres [21] that stimulate the tempoparietal junction [22]. This area is similar to the parietal-insular-vestibular area found in monkeys by Grüsser et al. [23, 24] which has neurons that respond to somesthetic stimulation, especially from the neck (apart from visual and vestibular inputs). It has been postulated that GCS impacts these neurons, supplying data to the central nervous system regarding the position of the head and the trunk in space [23, 24]. Therefore, GCS might improve the balance ability in the context of vestibular deficits [25], neglect patients [26] and in fixed simulators, where there is a lack of vestibular information (e.g., [27]). Previous research has found that reduction of head movements is related to less SAS symptoms [27, 28], finding that GCS reduces these head movements. In this avenue, it has been proposed that GCS reduces SAS symptoms through an improvement of the orientation of the head and trunk, which results in a reduction of movements produced by the imposed motion in simulator [27, 28]. This improvement of orientation perception has been supported by studies with galvanic vestibular stimulation (GVS), a similar technique to GCS where the electrodes are usually placed on the mastoid process. These studies found that GVS decrease MS [29, 30] and SAS [31] due to a reduction of sensory reliability of the vestibular system [29, 30], or a reconnection between visual cues and external vestibular cues [31], in line with the assumptions of the sensory conflict theory. Additionally, evidence suggests that the administration of GCS improves driving performance variables [27], such as faster speed in a high-speed turning maneuver. Faster speed was negatively correlated with SAS symptoms. The authors concluded that these findings can be interpreted as a mere increase in speed owing to less SAS symptoms, taking into account the high requirement of the driving task.

The mitigation of SAS could come from other sources apart from the body balance account (i.e., an improvement of body balance to mitigate SAS), for example, distraction from sickness symptoms (e.g., stomach discomfort, eyestrain). As Reason and Brand [4] remarked, individuals are less likely to experience MS when directing attention toward external events than when they focus on reporting their sensations. The observed attentional disengagement account is based on previous evidence. For example, some studies have approached the mitigation of SAS by employing different distractors. Gálvez-García et al. [32] provided tactile stimulation to the quadriceps (TSQ), avoiding the stimulation of muscles that could provide somatosensory information to the central nervous system about the position of the head or the trunk in space. Thus, the reduction of SAS with this stimulation would be likely due to an attentional disengagement from the symptoms. Furthermore, they measured head postural stability (head sway) to reduce the possibility that postural adjustment fully accounted for the mitigated SAS symptoms, in line with previous studies where vibrotactile stimulation did not affect body balance [33]. The authors found a significant reduction of SAS symptoms when TSQ was provided, without an increase in body balance and no adverse impact on driving performance variables. This finding reinforces the idea that the distraction of the participants from the symptoms without body balance improvement could reduce SAS, consistent with previous research where explicit mental distraction was effective in reducing SAS [28]. However, they did not measure any index to corroborate whether mental distraction impacted on body balance. In addition, this attentional disengagement account might explain, for example, why different types of stimulation, like acupuncture [34] or pleasant odors [20], mitigate SAS. Thus, the existing body of literature demonstrates that SAS is a complex phenomenon, which can be alleviated by modifying body balance factors and other processes such as attention.

Recent findings have proposed that a combination of different countermeasures could decrease MS and SAS more effectively than the administration of either vestibular or cognitive distractor countermeasures in isolation. In this avenue, Bos [17] found that the combination of head vibration and mental distraction was more effective at mitigating MS (39% reduction of MS symptoms) than vibration and mental distraction in isolation (25% and 19%, respectively). The authors conclude that both countermeasures distract participants from their symptoms, although vibration applied to the head might affect vestibular balance. Nevertheless, body balance was not measured to corroborate this hypothesis. Furthermore, their study was not performed with a driving simulator, whereby this reduction might not be evident in multisensory driving tasks where subjects must be focused on driving maneuvers. In a similar vein, Gálvez-García et al. [27] studied the combination of GCS and auditory stimulation. The last one mitigated SAS by improving balance similarly to GCS [28]. The additive effect of GCS and auditory stimulation was more effective at reducing SAS (73% reduction as compared to a baseline condition, where no stimulation was applied) than both countermeasures applied in isolation (50% and 48% reduction for GCS and auditory stimulation, respectively). Both stimulations improved body balance (especially when they were presented in conjunction), with an improvement in the required driving task (i.e., high speed when driving around a curve). Conversely, the combination of seat vibration and airflow was not effective at decreasing SAS because the null effect of seat vibration to mitigate SAS [35].

All in all, previous evidence indicates that the use of simultaneous techniques to reduce SAS—specifically, those targeting the body balance account [27] and the attentional disengagement account of SAS [32]–is more effective than their implementation in isolation. However, the combination of techniques from these two accounts has not been tested. This is important for two reasons. First, there is sparse literature regarding the exclusivity and additivity of techniques which mitigate SAS, and this study will provide further evidence for their use in combination. Second, it is fundamental to test whether techniques that have high effectiveness in reducing SAS by improving body balance (e.g., GCS), which also have evidenced positive effects on driving performance variables [27], can further reduce SAS along with other distracting techniques. In this way, techniques that have shown to impact body balance could be combined in the future with multiple distractors that have been tested in isolation, such as pleasant odors [20] and relaxing pleasant music [19].

Following the aforementioned premises, in the present study, we aimed to determine whether two techniques that have proved to be effective at decreasing SAS in isolation—GCS [27, 28, 36, 37] and TSQ [20]–have an additive effect when deployed simultaneously to decrease SAS. Furthermore, as previous studies have shown that an improvement in body balance (i.e., reduction of head movements produced by the imposed motion) is a crucial component when explaining the reduction of SAS by means of GCS [27, 29], we measured the head sway in all the conditions. Head sway was used during the simulator experience as an index of body balance, in line with previous research that highlighted the utility of this technique as a measure accounting for the variability of body balance when in the simulator [27, 28]; head balance is positively associated with SAS symptoms. As previously noted, studies with GCS (e.g., [27]) have shown evidence of improved driving performance (i.e., faster speed in curves), whereas no adverse effects have been revealed when using TSQ (e.g., [32]). However, in this latter, the participants were able to adjust their speed on the curves to perform this maneuver efficiently and thus, reducing the differences in driving performance between the TSQ and the baseline conditions. To address this concern in the current study, we measured driving performance variables in a demanding driving task (i.e., high-speed turning maneuver) to prevent the participants from reducing the speed during the driving task [27]. We anticipate that the combined administration of GCS and TSQ will be more effective at reducing SAS than their administration in isolation with an improvement of the driving performance. Importantly, we do not expect body balance to be modulated when TSQ is deployed, in line with previous research [32].

## Material and methods

### Participants

Forty healthy adults (mean age: 23.1 ± 3.4 years old, age range: 18–34 years old, 20 women) were recruited by intentional sampling. This sample was suggested by an *a priori* power analysis for medium effect size (*f*^2^ = 0.25) at power = 0.95 and α = 0.05 (*F* test family, ANOVA: Repeated measures, within-between interaction; G*Power version 3.1 [38]). In line with Gálvez-García et al. [27], before starting the experiment, the participants were asked to respond to the Motion Sickness Susceptibility Questionnaire (MSSQ) [39]. Individuals with MSSQ scores equal to 0 and higher than 65 (75^th^ percentile) were not included in the sample given their disinclination and propensity towards experiencing MS, respectively. The MSSQ mean score of the final sample was 40.7 ± 13.0. All participants were right-handed—assessed by the Spanish version of the Edinburgh Handedness Inventory [40]–and self-reported normal or corrected to normal vision. Inclusion criteria were as follow: i) being in a normal state of health (i.e., no cold and no chronic diseases), ii) have driven at least 3,000 km during the twelve months, iii) report normal vestibular function (Romberg’s test of vestibular dysfunction), iv) not use a pacemaker or a hearing aid, v) no history of vestibular vertigo, and vi) not being under medication that affects their driving performance. Moreover, the participants were asked to abstain from consuming alcoholic substances and caffeinated drinks for 24 hours before the experiment. Participants were informed that they could withdraw from the experiment with no negative consequences. All participants gave their signed written informed consent. This study was approved by the local Research Ethics Committee of the *Universidad de La Frontera* (N°046/20), and was carried out in compliance with the principles of the Declaration of Helsinki [41].

### Apparatus and stimuli

During the experiment, the participants were sitting in an adjustable car seat at a distance of 3.5 m from a screen (dimensions: 2.0 m wide × 1.4 m high; field-of-view: 31° horizontally, 24° vertically), on a fixed-base simulator. The experiment room was temperature-controlled (mean temperature = 23 ± 1.4°C). Open-source driving simulator software OpenDS Pro Complete version 5.0 was used to design a specific scenario and to render a driver’s front view. Participants controlled the car using a steering wheel, pedals and a gearbox. The simulated environment consisted of a 24.6 km flat route through an urban environment, where participants had to negotiate 27 curves to the left and 27 to the right (50% of gradual turn and 50% of sharp 90° turns). Gradual turns consisted of a 70 m entry, a 140 m curve and a 70 m exit, summing up a total of 250 m. The sharp turns were designed on a T-junction, with a 40 m lead-in and a 40 m curve, summing up 80 m in total. Between the curves, there were straight sections with a distance between 200 and 300 m. A high-speed digital camera (S-MOTION), placed above the screen in the middle, recorded head movements. Data processing of head movements was performed using an algorithm in MatlabR2016a software [42], where movements of the nose tip were measured in pixels along the X- and Y-axes.

TSQ was delivered by a small metallic rod of 1.5 mm (TSD190 tactile stimulator connected to STM100C module of Biopac MP160) with pulses of 0.5 s during all the curves followed a previously tested protocol [32, 43, 44]. Equally, a similar protocol to that used in previous studies (e.g., [27, 45]) was used to provide the GCS. Two electrodes (2.5 cm^2^) were placed bilaterally on top of the sternocleidomastoid muscles (3 to 4 cm below the mastoid process). The current output was provided at an intensity ranging from 0.6 to 1.3 mA, using the STMISOLA stimulus isolator (Biopac) following a similar procedure described by Reed-Jones et al. [37, 45] and Gálvez-García et al. [17, 19]. This output was set prior to the conduction tests, increasing by 0.05 mA until the participant performs small head movements. Finally, the output was adjusted to double the threshold defined for all subjects in line with previous research [27, 28, 46] where it has been pointed out that this procedure provided a comfortable and suitable stimulation strength. It should be noted that the software delivering GCS was programmed to provide a maximum of 200 mJ as a safety precaution (more than 300 mJ is dangerous, according to the device instruction manual). Finally, the total scores of the Simulator Sickness Questionnaire (SSQ) [47] were used to measure SAS, where 16 symptoms were rated in a scale ranging from 0 ("none") to 3 ("severe").

### Procedure

The experimental conditions were the following: i) baseline condition, where no stimulation was delivered; ii) GCS condition, where GCS was delivered from 40 m before a curve to the end of the curve; iii) TSQ condition, where TSQ was delivered from 40 m before a curve to the end of the curve; iv) GCS + TSQ condition, where GCS and TSQ were delivered simultaneously from 40 m before a curve to the end of the curve. The participants performed a familiarization session for 5 minutes at the beginning of each experimental condition. The tappers and the GCS electrodes were placed at the beginning of all conditions to maintain the same set-up across the different experimental conditions and to rule out the mere presence of the electrodes and tappers as an explicatory factor of the data. The order of the experimental conditions was counterbalanced following a Latin-square design to control carry-over/adaptation effects. To control sickness accumulation, the experimental conditions were performed with four days of difference to avoid the persistence of the symptoms [48].

To prevent participants from reducing their speed throughout the circuit, we instructed the participants to drive at a speed ranging from 80 to 90 km/h on the right hand side of the circuit during the leading. Moreover, the participants were instructed not to reduce their speed to less than 70 km/h during the curves. Leading and curves with speed less than 80 km/h and 70 km/h respectively were excluded from the analyses (three lead-ins and four curves among all participants).

### Data analyses

We used RStudio (version 1.1.383; RStudio Team [49]), for all our analyses. In line with previous research (e.g., [27, 28, 32]), the SSQ total score, the head sway (i.e., the standard deviation of head movements in pixels along the X- and Y-axes during the curves of the circuit) and two driving performance variables were considered as dependent variables. The driving performance variables corresponded to the average speed (km/h) during the curves, and the steering wheel variability (*SD* of steering wheel position) during the curves. Both slower speed and less steering wheel movements have been associated with more conservative driving [50]. Whereas the speed has been negatively correlated to SAS [27], the relationship between steering movements and SAS has not been directly tested. Thus, we included steering wheel movements as an additional driving performance variable. We computed linear mixed-effects models (LME) for each dependent variable using the *nlme* [51] and the *lme4* [52] packages. Mixed-effects modelling offers a series of advantages when it comes to repeated-measures designs as compared to, for instance, ANOVA tests. Most relevant, it allows researchers to take into account all the potential factors that might contribute to the explanation of a dataset simultaneously (i.e., fixed and random effects) and provides enhanced statistical power. For more details on the advantages of mixed-effects modelling as compared to more traditional methods, we refer the interested reader to Baayen et al. [53]. It should be noted that none of the dependent variables was normally distributed, as indicated by the Shapiro-Wilk normality test (SSQ total score: *W* = 0.954, *p* < 0.001; Head sway along the X axis: *W* = 0.920, *p* < 0.001; Head sway along the Y axis: *W* = 0.976, *p* = 0.006; Average speed: *W* = 0.966, *p* < 0.001; Steering wheel variability: *W* = 0.974, *p* = 0.004). Following Wibirama et al. [54], we performed non-parametric tests. However, given the robustness and advantages of the mixed-effects modelling [53], and the fact that comparable results were found when using non-parametric test, here we report the results derived from the LME models. The results of the non-parametric tests (i.e., Friedman test considering Kendall’s *W* as an estimate of effect size, and Wilcoxon signed-rank tests for multiple comparisons) are reported in the S1 Data. All the LME models included the factor "condition" as fixed effect (i.e., baseline vs. GCS vs. TSQ vs. GCS + TSQ), and "participants" as a random effect to account for a more general estimate of the fixed effect by taking into account the stochastic variability in the dataset [55]. Following a model comparison approach [56], we contrasted the computed LME models, considering the model with the lowest Akaike information criterion (AIC) as the best fitting model [57]; we computed the exponential of the difference between the models’ AIC to account for the relative likelihood of a given model [AIC_RL_ = exp(ΔAIC/2)] (e.g., [57]). Thus, for each dependent variable, we compared the model including the fixed factor (condition) against a null model including no fixed effect. We performed likelihood ratio tests to extract the *p*-values. As the fixed factor condition included four levels, we carried out multiple comparisons by using the *lsmeans* package [58]. We selected the Tukey method for the adjustment of *p*-values to reduce the probability of type 1 error. Moreover, we used the *piecewise SEM* package [59] to compute the marginal and conditional *R*^2^ in order to account for the proportion of variance explained by the fixed effect (*R*^2^_m_) and by both the fixed and random effects (*R*^2^_c_), respectively, on each LME model. For each dependent variable, we reported the mean and *SD* per condition. Furthermore, we computed Spearman’s rho correlation coefficients (*r*_*s*_) to account for the association between the SSQ total score, the head sway along the X- and Y-axes, and the driving performance variables (average speed and steering wheel variability) per condition. It should be pointed out that some previous studies (e.g., [37]) have analyzed (apart from the SSQ total scores) the output of the SSQ by grouping the different symptoms into three groups (i.e., nausea, oculomotor and disorientation; following Kennedy et al. [47]) However, several of the SSQ items are represented on multiple subscales which could lead to family-wise error rate, decreasing the reliability of the results. Thus, LME models and non-parametric tests for the three SSQ sub-scores (following the same procedure described in the main analysis) are reported in the S1 Data.

## Results

### SSQ scores

**Fig 1:**
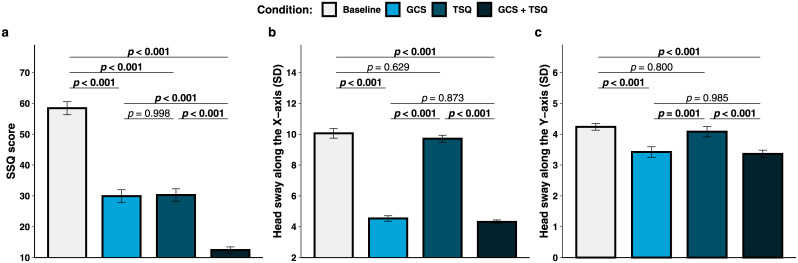
(a) SSQ score, (b) head sway along the X-axis, and (c) head sway along the Y-axis per condition. Error bars represent standard error of the mean.

A significant main effect of condition was found on the SSQ total scores (see Fig 1A), χ^2^(3) = 192.57, *p* < 0.001, AIC_RL_ > 100, *R*^2^_m_ = 0.668, *R*^2^_c_ = 0.767. Multiple comparisons revealed that lower SSQ scores were reported in the GCS + TSQ condition (12.4 ± 6.6) than in the baseline [58.4 ± 13.5, *t*(39) = 20.787, *p* < 0.001], the GCS [29.9 ± 13.2, *t*(39) = 7.901, *p* < 0.001] and the TSQ [30.3 ± 12.6, *t*(39) = 8.070, *p* < 0.001] conditions. The SSQ scores were also significantly lower for both the GCS [*t*(39) = 12.886, *p* < 0.001] and the TSQ [*t*(39) = 12.717, *p* < 0.001] conditions as compared to the baseline condition. Instead, the scores did not differ significantly between the GCS and the TSQ conditions [*t*(39) = -0.169, *p* = 0.998]. In short, the delivery of GCS + TSQ was more effective at decreasing the SAS symptoms than their isolated presentation.

### Head sway

The main effect of condition was significant on the head sway along the X-axis (see Fig 1B), χ^2^(3) = 257.12, *p* < 0.001, AIC_RL_ > 100, *R*^2^_m_ = 0.796, *R*^2^_c_ = 0.823. Multiple comparisons revealed higher head sway for the baseline condition (10.1 ± 2.0) than for the GCS [4.5 ± 1.1, *t*(39) = 18.857, *p* < 0.001] and the GCS + TSQ [4.3 ± 0.8, *t*(39) = 19.614, *p* < 0.001] conditions. Furthermore, higher head sway along the X-axis was observed within the TSQ condition (9.7 ± 1.4) as compared to the GCS [*t*(39) = -17.659, *p* < 0.001] and the GCS + TSQ conditions [*t*(39) = 18.416, *p* < 0.001]. No significant differences were found between the baseline and the TSQ conditions [*t*(39) = 1.198, *p* = 0.629], nor between the GCS and the GCS + TSQ conditions [*t*(39) = 0.757, *p* = 0.873]. A significant main effect of condition was also found on the head sway along the Y-axis (see Fig 1C), χ^2^(3) = 35.554, *p* < 0.001, AIC_RL_ > 100, *R*^2^_m_ = 0.158, *R*^2^_c_ = 0.392. Higher head sway was observed within the baseline condition (4.2 ± 0.7) as compared to the GCS [3.4 ± 1.1, *t*(39) = 4.716, *p* < 0.001] and the GCS + TSQ [3.4 ± 0.8, *t*(39) = 5.069, *p* < 0.001] conditions. Moreover, head sway along the Y-axis was higher for the TSQ condition (4.1 ± 1.1) than for the GCS [*t*(39) = -3.807, *p* = 0.001] and the GCS + TSQ [*t*(39) = 4.161, *p* < 0.001] conditions. Instead, the differences between the baseline and the TSQ conditions [*t*(39) = 0.909, *p* = 0.800], and between the GCS and the GCS + TSQ conditions [*t*(39) = 0.354, *p* = 0.985] did not reach significance. To sum up, the administration of GCS—both in isolation and combined with TSQ—yielded to lower head sway along the X-axis and the Y-axis, whereas the administration of TSQ did not affect the head sway.

### Driving performance variables

**Fig 2:**
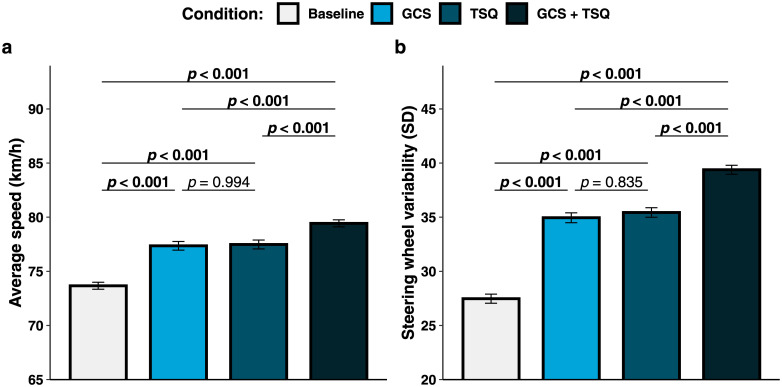
(a) Average speed, and (b) steering wheel variability per condition. Error bars represent standard error of the mean.

The main effect of condition was significant on the average speed (see Fig 2A), χ^2^(3) = 102.58, *p* < 0.001, AIC_RL_ > 100, *R*^2^_m_ = 0.455, *R*^2^_c_ = 0.547. Multiple comparisons showed that the speed was higher for the GCS + TSQ condition (79.4 ± 2.0 km/h) than for the baseline [73.7 ± 2.0 km/h, *t*(39) = -12.211, *p* < 0.001], the GCS [77.3 ± 2.5 km/h, *t*(39) = -4.493, *p* < 0.001] and the TSQ [77.5 ± 2.6 km/h, *t*(39) = -4.139, *p* < 0.001] conditions. The average speed was also higher for the GCS [*t*(39) = -7.818, *p* < 0.001] and the TSQ [*t*(39) = -8.073, *p* < 0.001] conditions as compared to the baseline condition. Instead, the speed within the GCS and the TSQ conditions did not differ significantly [*t*(39) = -0.254, *p* = 0.994]. As for the steering wheel variability, a significant main effect of condition was retrieved (see Fig 2B), χ^2^(3) = 203.92, *p* < 0.001, AIC_RL_ > 100, *R*^2^_m_ = 0.717, *R*^2^_c_ = 0.747. Multiple comparisons revealed higher variability in the GCS + TSQ condition (39.4 ± 2.6) as compared to the baseline [27.5 ± 2.6, *t*(39) = -20.519, *p* < 0.001], the GCS [34.9 ± 2.9, *t*(39) = -7.638, *p* < 0.001], and the TSQ [35.4 ± 2.8, *t*(39) = -6.799, *p* < 0.001] conditions. The steering wheel variability was also higher in the GCS [*t*(39) = -12.881, *p* < 0.001] and the TSQ [*t*(39) = -13.721, *p* < 0.001] conditions than in the baseline condition. In contrast, the difference between the GCS and the TSQ conditions was not significant [*t*(39) = -0.840, *p* = 0.835]. In brief, the average speed and the steering wheel variability were higher when presented with GCS + TSQ than when these techniques were not administered or were displayed separately.

### Correlations

The SSQ scores were positively correlated with the head sway. Along the X-axis, the correlation was moderate for the baseline, the GCS and the TSQ conditions, and strong for the GCS + TSQ condition. Along the Y-axis, the correlation was strong for the GCS condition, moderate for the TSQ and the GCS + TSQ conditions, and weak (albeit significant) for the baseline condition. Furthermore, the SSQ scores and the average speed were negatively associated. The correlation was strong for the baseline and the GCS + TSQ conditions, moderate for the GCS condition, and weak (although significant) for the TSQ condition. Moreover, the SSQ scores were negatively associated with the steering wheel variability. The correlation was strong for the baseline and the GCS + TSQ conditions, and weak (but significant) for the GCS and the TSQ conditions. The Spearman correlation coefficients are presented in Table 1.

**Table 1:** Spearman’s rho correlation coefficients (*r*_*s*_) for the association between SSQ score, head sway, and driving performance variables per condition.

SSQ score	Head sway along the X-axis	Head sway along the Y-axis	Average speed	Steering wheel variability
Baseline	*r*_*s*_(38) = 0.57, *p* < 0.001	*r*_*s*_(38) = 0.45, *p* = 0.004	*r*_*s*_(38) = -0.66, *p* < 0.001	*r*_*s*_(38) = -0.63, *p* < 0.001
GCS	*r*_*s*_(38) = 0.58, *p* < 0.001	*r*_*s*_(38) = 0.60, *p* < 0.001	*r*_*s*_(38) = -0.55, *p* = 0.013	*r*_*s*_(38) = -0.38, *p* = 0.015
TSQ	*r*_*s*_(38) = 0.58, *p* < 0.001	*r*_*s*_(38) = 0.51, *p* < 0.001	*r*_*s*_(38) = -0.39, *p* < 0.001	*r*_*s*_(38) = -0.39, *p* = 0.013
GCS + TSQ	*r*_*s*_(38) = 0.62, *p* < 0.001	*r*_*s*_(38) = 0.53, *p* < 0.001	*r*_*s*_(38) = -0.62, *p* < 0.001	*r*_*s*_(38) = -0.76, *p* < 0.001

## Discussion

The main aim of this study was to test the effectiveness of GCS and TSQ at mitigating SAS during a driving task, both when delivered in isolation and in conjunction. Concerning the application of techniques in isolation, our data confirmed that GCS alleviates SAS with a reduction of 48.8% of symptoms as compared to the baseline condition (where no stimulation was delivered), consistent with previous research [2, 27, 28, 36]. This reduction of symptoms is related to an improvement of postural adjustment (less head sway for the X- and Y-axes) according to body balance account [27]. Furthermore, our data aligned with previous evidence showing that TSQ mitigates SAS [32], with a reduction of 48.1% of as compared to the baseline condition. Importantly, this reduction does not depend on an improvement in balance ability (i.e., no differences between the baseline and TSQ condition regarding head sway). We argue that TSQ produces a distraction from being aware of their symptoms, in line with attentional disengagement account [34]. Besides, other attentional factors could coexist, such as an attenuation of the sensation of motion by distraction reducing the conflict between the visual and the other senses [46]. The importance of corroborating a measure that decreases SAS without impacting balance ability should be noted. This finding aligns with previous studies suggesting that impairment of balance ability is not the only explanatory factor of MS (e.g., [60]). In fact, although GCS showed significantly less head sway for the X- and Y-axes and TSQ did not, both sources of stimulation decreased SAS in a similar proportion. More generally, this provides additional evidence that SAS is not a simple phenomenon that only affects body balance factors, but also other processes, such as attention, in line with previous research on SAS [20] and MS [17].

The idea that SAS relates to multiples factors is reinforced by the drastic decrease of SAS symptoms when GCS and TSQ are applied in conjunction (78.8%), as compared to the baseline condition. Importantly, the SSQ total scores for the GCS and the TSQ conditions were 29.9 and 30.3, respectively, which according to Webb et al. [61], still represents discomfort (i.e., scores > 20). However, for the combined administration of GCS and TSQ, the SSQ total score was 12.4, which suggests that the participants barely experienced any discomfort. It should be remarked that the reduction of SAS for the GCS + TSQ condition is not related to a greater improvement of body balance compared with the GCS condition (i.e., no significant difference regarding head sway along the X- and Y-axes). Thus, our results support the idea that the combination of techniques that impact on body balance and attentional factors is an effective method to alleviate SAS.

The results of driving performance also support the application of GCS and TSQ separately and, especially, in conjunction. Both techniques enhanced the success of performance on the proposed demanding driving task (i.e., speed constraints), such that faster speeds led to more steering movements [50], in line with previous studies where GCS was tested with a similar demanding driving task [27]. This improvement in driving performance was higher when GCS and TSQ are applied in conjunction. Moreover, we found a positive correlation between speed and steering movements with SAS in all conditions. This confirms that SAS produces a more conservative driving style owing to SAS [50]. It should be pointed out that previous research did not find an improvement in driving performance when using TSQ [32]. This could be because participants could adjust their speed on the curves to perform this maneuver efficiently, reducing the differences in driving performance between conditions. In line with our expectations, the inclusion of a more demanding task is the key to capture differences regarding driving variables (i.e., speed and steering movements) between the TSQ and the baseline conditions. Furthermore, it should be noted that GCS and TSQ could have generated some interference with driving because of multiple sources of stimulation. However, this seems not to have been the case, as an improvement in driving performance was observed. Overall, our results reveal that GCS and TSQ—especially when presented in conjunction—improve driving performance, which is a fundamental point for their recommendation for future interventions aiming to reduce SAS.

Some consideration should be noted about the processes by which GCS and TSQ reduce SAS. Our results suggest that the reduction of symptoms by TSQ is due to a distraction from symptoms [32], as supported by the null effect in body balance. Furthermore, our data show that the reduction of SAS by GCS is mainly related to the improvement of body balance—as indexed by reduced head movements—being the head sway positively correlated with SAS symptoms. More concretely, GCS could reduce SAS symptoms improving the orientation of the head and trunk, resulting in a reduction of movements produced by the imposed motion in simulators [27, 28]. In any case, it should be noted that our pattern of improvement of body balance could be related to different theories about the ateology of SAS. On the one hand, the improvement of orientation perception could be one of the key factors to reduce SAS, and thus our pattern of data could support the vertical subjective theory [8], previous studies with GVS [29–31] and more generally, previous work where it has been highlighted that GCS improves orientation perception [23, 24]. On the other hand, the theory of postural instability [9] states that SAS is caused by an inability to maintain a stable pattern of movement, whether this stability is slowly degraded or outright lost. Our data do not conflict with the latter theory. Our findings support that GCS improves body balance (and consequently reduces SAS). Thus, GCS could prevent the inability to maintain a stable pattern of movement by improving the orientation of the head. However, although not directly addressed, it has been suggested that GCS could additionally distract the participants from the symptoms (i.e., from being conscious of their sickness; e.g., [28]). Thus, although our data mostly support that GCS decreases SAS by an improvement in body balance, a distraction from the symptoms may occur with GCS. However, to the best of our knowledge, there is no empirical data that support this idea. Further, our results support the idea that SAS is not a simple phenomenon that only affected body balance factors, but also other processes, such as attention. These attentional processes are not restricted to disengagement from the symptoms. For example, Seno et al. [62, 63] found that attention could moderate the severity of the induced self-motion sensation caused by visual stimulation, and limits the conflict between visual and the other senses. In this vein, Wei et al. [64] found that allocating less attention to central vision produces less MS symptoms. Thus, the attentional disengagement account could coexist with other attentional factors.

All in all, our study confirms that GCS and TSQ are effective to reduce SAS in a fixed-base simulator when applied in isolation. More importantly, the combination of GCS and TSQ has proved to be an especially effective countermeasure at mitigating SAS because of their impact on body balance and attentional components, respectively. Furthermore, the joint and individual use of GCS and TSQ improves driving performance. Finally, identifying attention-related factors which mitigate SAS is relevant, as a series of stimulating devices can be implemented into the driving simulation set-up. However, care should be taken regarding the intensity of the source of distraction, as high levels of distraction might lead to a significant driving performance impairment. Future research is needed to generalize our results, for instance, by testing different driving scenarios and populations such as elderly drivers who might be more prone to SAS [65]. In addition, the electrodes and tappers for GCS and TSQ, respectively, were placed in the baseline condition to rule out the mere presence of the electrodes and tappers as an explicatory factor of the results. Future research could compare the baseline condition used in the current study with a condition where no electrodes and tappers to study this manipulation as a passive technique to distract from the SAS symptoms. Other future research should consider the strategic differences between participants in head motion (which could play a role in SAS). Thus, a six-degrees-of-freedom measurement of head motion would allow the separation of rotation/translation of the head. Further, we encourage the combination of GCS and TSQ with other techniques that have proved to be effective at alleviating SAS, such as progressive exposure to the simulator and habituation [66–68], auditory stimulation [28], the generation of movements against centrifugal acceleration [69] and pleasant olfactory stimulation [20], among others. Moreover, future studies could address the comparison between demanding driving task with different speed constraints to generalize our results. Future studies might also use real-time and more objective measurement of SAS, such as forehead skin conductance [70]. Finally, more studies focused on SAS using a broader and multicausal perspective are needed. In this line, Lackner [71] showed how MS is a complex syndrome that transgresses nausea and vomiting and could cause sopite syndrome [72, 73]. This syndrome is featured by the profound drowsiness and persistent fatigue following motion stimulation. In this vein, different distractive techniques could countermeasure this SAS-related syndrome.

## Supporting information

S1 Data (DOC)
