# Supplementary material for: Joint and individual effectiveness of galvanic cutaneous stimulation and tactile stimulation at decreasing Simulator Adaptation Syndrome
Source: PLoS One. 2020 Oct 15;15(10):e0240627. doi: 10.1371/journal.pone.0240627 (PMC7561193; doi:10.1371/journal.pone.0240627)
Supplement: S1 Data — (DOC) [file pone.0240627.s001.doc]

# Supplementary material

***SSQ sub-scales scores***

Following the same procedures described in the main manuscript, here we report the results of the three SSQ sub-scales (i.e., nausea, oculomotor, and disorientation; Kennedy et al., 1993) as a function of condition (see *Data analyses* in the main manuscript). The scores of the three SSQ sub-scales followed the same pattern of results than the SSQ total scores (see *Results* in the main manuscript).

***SSQ nausea.*** The main effect of condition was significant on the scores of the SSQ nausea sub-scale (see Fig S1A), χ^2^(3) = 135.48, *p* < 0.001, AIC_RL_ > 100, *R*^2^_m_ = 0.531, *R*^2^_c_ = 0.662. Multiple comparisons showed lower scores in the GCS + TSQ condition (11.4 ± 8.4) as compared to the baseline [50.8 ± 16.4, *t*(39) = 15.216, *p*< 0.001], the GCS [25.5 ± 13.4, *t*(39) = 5.441, *p*< 0.001] and the TSQ [24.3 ± 15.0, *t*(39) = 4.980, *p*< 0.001] conditions. Lower scores were also reported for both the GCS [*t*(39) = 9.775, *p*< 0.001] and the TSQ [*t*(39) = 10.236, *p*< 0.001] conditions as compared to the baseline condition. The scores did not differ significantly between the GCS and the TSQ conditions [*t*(39) = 0.461, *p* = 0.967].

# *SSQ oculomotor.* A significant main effect of condition was retrieved on the scores of the SSQ oculomotor sub-scale (see Fig S1B), χ^2^(3) = 156.48, *p* < 0.001, AIC_RL_ > 100, *R*^2^_m_ = 0.558, *R*^2^_c_ = 0.724. Multiple comparisons revealed that lower scores were reported in the GCS + TSQ condition (11.4 ± 7.1) than in the baseline [48.9 ± 14.0, *t*(39) = 17.537, *p*< 0.001], the GCS [25.6 ± 13.3, *t*(39) = 6.643, *p*< 0.001] and the TSQ [28.0 ± 12.7, *t*(39) = 7.794, *p*< 0.001] conditions. The scores were also lower for both the GCS [*t*(39) = 10.894, *p*< 0.001] and the TSQ [*t*(39) = 9..743, *p*< 0.001] conditions as compared to the baseline condition. No significant differences were found between the GCS and the TSQ conditions [*t*(39) = -1.151, *p* = 0.658].

# *SSQ disorientation.* The scores of the SSQ disorientation sub-scale were significantly modulated by condition (see Fig S1C), χ^2^(3) = 106.19, *p* < 0.001, AIC_RL_ > 100, *R*^2^_m_ = 0.478, *R*^2^_c_ = 0.535. Multiple comparisons revealed lower scores in the GCS + TSQ condition (8.7 ± 13.3) as compared to the baseline [53.6 ± 22.2, *t*(39) = 12.491, *p*< 0.001], the GCS [27.1 ± 17.0, *t*(39) = 5.132, *p*< 0.001] and the TSQ [25.7 ± 14.3, *t*(39) = 4.745, *p*< 0.001] conditions. The scores were also lower for both the GCS [*t*(39) = 7.359, *p*< 0.001] and the TSQ [*t*(39) = 7.746, *p*< 0.001] conditions than for the baseline condition. The scores for the GCS and the TSQ conditions did not differ significantly [*t*(39) = 0.387, *p* = 0.980].

**Fig S1. SSQ sub-scales scores: (a) nausea, (b) oculomotor, and (c) disorientation. Error bars represent standard error of the mean.**

***Non-parametric tests***

As indicated in the main manuscript, none of the dependent variables was normally distributed. This was also the case for the three SSQ sub-scales (nausea: *W* = 0.976, *p* = 0.006; oculomotor: *W* = 0.976, *p* = 0.006; disorientation: *W* = 0.976, *p* = 0.006). Whereas in the main manuscript we reported the results of linear mixed-effects models, here we report the results of the non-parametric tests (i.e., Friedman test considering Kendall’s *W* as an estimate of effect size and Wilcoxon signed-rank tests for multiple comparisons). Importantly, all variables followed the same pattern of results than those obtained when using linear mixed-effects models. The results are summarized in Table S1.

**Table S1. Results of non-parametric tests informing the main effect of condition on each dependent variable and multiple comparisons.**

| **Variable** | **Main effect of condition**  **(Friedman test and Kendall’s *W*)** | **Multiple comparisons** | |
| --- | --- | --- | --- |
|  |  | **Conditions** | **Wilcoxon signed-rank tests** |
| SSQ total score | χ^2^(3) = 98.330, *p*< 0.001,  Kendall’s *W* = 0.819 | Baseline vs. GCS | *W* = 820.0, *p*< 0.001 |
|  |  | Baseline vs. TSQ | *W* = 778.5, *p*< 0.001 |
|  |  | Baseline vs. GCS + TSQ | *W* = 820.0, *p*< 0.001 |
|  |  | GCS vs. TSQ | *W* = 232.5, *p* = 1.000 |
|  |  | GCS + TSQ vs. GCS | *W* = 644.5, *p*< 0.001 |
|  |  | GCS + TSQ vs. TSQ | *W* = 756.0, *p*< 0.001 |
| SSQ nausea | χ^2^(3) = 82.092, *p*< 0.001,  Kendall’s *W* = 0.684 | Baseline vs. GCS | *W* = 689.5, *p*< 0.001 |
|  |  | Baseline vs. TSQ | W = 588.0, p < 0.001 |
|  |  | Baseline vs. GCS + TSQ | *W* = 780.0, *p*< 0.001 |
|  |  | GCS vs. TSQ | *W* = 178.0, *p* = 1.000 |
|  |  | GCS + TSQ vs. GCS | *W* = 513.0, *p*< 0.001 |
|  |  | GCS + TSQ vs. TSQ | *W* = 560.5, *p*< 0.001 |
| SSQ oculomotor | χ^2^(3) = 91.290, *p*< 0.001,  Kendall’s *W* = 0.761 | Baseline vs. GCS | *W* = 737.0, *p*< 0.001 |
|  |  | Baseline vs. TSQ | *W* = 662.0, *p*< 0.001 |
|  |  | Baseline vs. GCS + TSQ | *W* = 820.0, *p*< 0.001 |
|  |  | GCS vs. TSQ | *W* = 64.5, *p* = 0.149 |
|  |  | GCS + TSQ vs. GCS | *W* = 624.5, *p*< 0.001 |
|  |  | GCS + TSQ vs. TSQ | *W* = 606.0, *p*< 0.001 |
| SSQ disorientation | χ^2^(3) = 73.174, *p*< 0.001,  Kendall’s *W* = 0.610 | Baseline vs. GCS | *W* = 535.5, *p*< 0.001 |
|  |  | Baseline vs. TSQ | *W* = 591.5, *p*< 0.001 |
|  |  | Baseline vs. GCS + TSQ | *W* = 798.0, *p*< 0.001 |
|  |  | GCS vs. TSQ | *W* = 122.0, *p* = 1.000 |
|  |  | GCS + TSQ vs. GCS | *W* = 443.5, *p*< 0.001 |
|  |  | GCS + TSQ vs. TSQ | *W* = 541.5, *p*< 0.001 |
| Head sway along the X-axis | χ^2^(3) = 91.77, *p*< 0.001,  Kendall’s *W* = 0.765 | Baseline vs. GCS | *W* = 819, *p*< 0.001 |
|  |  | Baseline vs. TSQ | *W* = 269, *p* = 1.000 |
|  |  | Baseline vs. GCS + TSQ | *W* = 820, *p*< 0.001 |
|  |  | GCS vs. TSQ | *W* = 0, *p*< 0.001 |
|  |  | GCS + TSQ vs. GCS | *W* = 508, *p* = 1.000 |
|  |  | GCS + TSQ vs. TSQ | *W* = 819, *p*< 0.001 |
| Head sway along the Y-axis | χ^2^(3) = 39.09, *p*< 0.001,  Kendall’s *W* = 0.326 | Baseline vs. GCS | *W* = 713, *p*< 0.001 |
|  |  | Baseline vs. TSQ | *W* = 460, *p* = 1.000 |
|  |  | Baseline vs. GCS + TSQ | *W* = 735, *p*< 0.001 |
|  |  | GCS vs. TSQ | *W* = 116, *p*< 0.001 |
|  |  | GCS + TSQ vs. GCS | *W* = 444, *p* = 1.000 |
|  |  | GCS + TSQ vs. TSQ | *W* = 656, *p*= 0.004 |
| Average speed | χ^2^(3) = 73.174, *p*< 0.001,  Kendall’s *W* = 0.565 | Baseline vs. GCS | *W* = 43, *p*< 0.001 |
|  |  | Baseline vs. TSQ | *W* = 11, *p*< 0.001 |
|  |  | Baseline vs. GCS + TSQ | *W* = 3, *p*< 0.001 |
|  |  | GCS vs. TSQ | *W* =390, *p* = 1.000 |
|  |  | GCS + TSQ vs. GCS | *W* = 134, *p*< 0.001 |
|  |  | GCS + TSQ vs. TSQ | *W* = 171, *p*= 0.006 |
| Steering wheel variability | χ^2^(3) = 90.51, *p*< 0.001,  Kendall’s *W* = 0.754 | Baseline vs. GCS | *W* = 4, *p*< 0.001 |
|  |  | Baseline vs. TSQ | *W* = 0, *p*< 0.001 |
|  |  | Baseline vs. GCS + TSQ | *W* = 0, *p*< 0.001 |
|  |  | GCS vs. TSQ | *W* =313, *p* = 1.000 |
|  |  | GCS + TSQ vs. GCS | *W* = 47, *p*< 0.001 |
|  |  | GCS + TSQ vs. TSQ | *W* = 45, *p*< 0.001 |
